# Enhancing maize resilience to drought stress: the synergistic impact of deashed biochar and carboxymethyl cellulose amendment

**DOI:** 10.1186/s12870-024-04843-w

**Published:** 2024-02-27

**Authors:** Subhan Danish, Zuhair Hasnain, Khadim Dawar, Shah Fahad, Adnan Noor Shah, Saleh H. Salmen, Mohammad Javed Ansari

**Affiliations:** 1 Department of Soil Science, Faculty of Agricultural Sciences and Technology, Bahauddin Zakariya University, Multan, Punjab Pakistan; 2 Department of Agronomy, Pir Mehr Ali Shah Arid Agriculture University, Rawalpindi, Pakistan; 3 Department of Soil and Environmental Science, the University of Agriculture Peshawar, Peshawar, Pakistan; 4 Department of Agronomy, Abdul Wali Khan University Mardan, Mardan, Khyber Pakhtunkhwa 23200 Pakistan; 5 Department of Natural Sciences, Lebanese American University, Byblos, Lebanon; 6 Department of Agricultural Engineering, Khwaja Fareed University of Engineering and Information Technology Rahim Yar Khan, Rahim Yar Khan, Punjab 64200 Pakistan; 7 Department of Botany and Microbiology, College of Science, King Saud University, PO Box -2455, Riyadh, 11451 Saudi Arabia; 8 Department of Botany, Hindu College Moradabad (Mahatma Jyotiba Phule Rohilkhand University Bareilly), Moradabad, 244001 India

**Keywords:** Soil amendments, Crop resilience, Water retention, Plant performance, Environmental stress mitigation

## Abstract

Drought stress poses a significant challenge to maize production, leading to substantial harm to crop growth and yield due to the induction of oxidative stress. Deashed biochar (DAB) in combination with carboxymethyl cellulose (CMC) presents an effective approach for addressing this problem. DAB improves soil structure by increasing porosity and water retention and enhancing plant nutrient utilization efficiency. The CMC provides advantages to plants by enhancing soil water retention, improving soil structure, and increasing moisture availability to the plant roots. The present study was conducted to investigate the effects of DAB and CMC amendments on maize under field capacity (70 FC) and drought stress. Six different treatments were implemented in this study, namely 0 DAB + 0CMC, 25 CMC, 0.5 DAB, 0.5 DAB + 25 CMC, 1 DAB, and 1 DAB + 25 CMC, each with six replications, and they were arranged according to a completely randomized design. Results showed that 1 DAB + 25 CMC caused significant enhancement in maize shoot fresh weight (24.53%), shoot dry weight (38.47%), shoot length (32.23%), root fresh weight (19.03%), root dry weight (87.50%) and root length (69.80%) over control under drought stress. A substantial increase in maize chlorophyll a (40.26%), chlorophyll b (26.92%), total chlorophyll (30.56%), photosynthetic rate (21.35%), transpiration rate (32.61%), and stomatal conductance (91.57%) under drought stress showed the efficiency of 1 DAB + 25 CMC treatment compared to the control. The enhancement in N, P, and K concentrations in both the root and shoot validated the effectiveness of the performance of the 1 DAB + 25 CMC treatment when compared to the control group under drought stress. In conclusion, it is recommended that the application of 1 DAB + 25 CMC serves as a beneficial amendment for alleviating drought stress in maize.

## Introduction

Drought poses a significant threat to maize production, affecting yield and quality [1–3]. Maize is highly vulnerable to water stress during crucial growth stages, leading to stunted growth and reduced yields [4, 5]. This emphasizes the need for adaptive measures, such as water-efficient farming practices, to safeguard global food security [6].

Existing approaches seek to address the challenges posed by drought in maize production by promoting water-efficient farming methods. However, the widespread adoption of these solutions encounters significant obstacles [7–9]. On the other hand, considerable time and financial resources needed for researching and developing resilient maize varieties present accessibility challenges for farmers [10, 11].

Carboxymethyl cellulose (CMC) is a cellulose derivative, which is a modified form of cellulose, a naturally occurring polymer found in the cell walls of plants [12]. It plays a vital role in alleviating the impact of drought conditions in agriculture. As a derivative of water-soluble cellulose, CMC enhances soil water retention, thereby improving its ability to retain moisture. Whether applied to soil or used as a seed coating, CMC serves as a protective layer that helps seeds retain essential moisture and nutrients crucial for germination and early plant growth, particularly in regions prone to drought [12]. Moreover, CMC contributes to enhancing soil structure, preventing compaction, and facilitating improved water infiltration [13]. These attributes establish CMC as a valuable tool for bolstering crop resilience to water scarcity and supporting the implementation of sustainable agricultural practices in drought-affected areas [14].

Biochar, generated by the thermal breakdown of organic biomass, is a carbon-rich, porous substance utilized as a sustainable soil enhancer to enhance agricultural productivity and facilitate carbon sequestration [8, 15–17]. Deashed biochar (DAB) is biochar that has undergone a treatment to minimize or eliminate its ash content [18]. It plays a crucial role in drought conditions by improving water retention in the soil through its porous structure, serving as a reservoir for plant moisture. Additionally, biochar acts as a nutrient sponge, preventing nutrient leaching and ensuring essential elements are available to plants even in water-scarce environments [18]. Its incorporation into the soil also contributes to enhanced soil structure, promoting better water infiltration and mitigating the adverse effects of drought on plant growth [19].

The combined application of CMC and deashed biochar requires comprehensive investigations to identify their role in the drought-stress environment under the maize production system [20]. The study hypothesized that the combined application of CMC and deashed biochar would improve maize productivity as compared to their individual effects. The main objective of the study was to evaluate the Impact of CMC and deashed biochar on the growth, physiological, and yield attributes of the maize under drought normal and drought stress conditions.

## Materials and methods

### Preparation of biochar

Cotton sticks were used as a waste product in the biochar synthesis process, and they were pyrolyzed at a temperature of 440 °C for 120 min. The physical, chemical, and nutritional characteristics of the produced biochar were next evaluated. The biochar was then cooled, then it was grinded to a size of 2 mm and placed in storage for later use.

### Deashing of biochar

The initial step involved rinsing the raw biochar with deionized water to eliminate water-soluble ash constituents. This rinsing procedure comprised immersing the biochar in water and employing repetitive filtration (a total of six times) to distinguish the biochar from the liquid. Following the rinsing process, excess moisture was eliminated by drying the biochar. Subsequently, the dried biochar underwent sieving using a sieve with a mesh size of less than 2 mm to attain a consistent particle size distribution.

### Characterization of biochar

Gravimetric analysis was used to identify the content of the biochar in accordance with the approach outlined by [21]. In order to measure the biochar’s pH [22] and electrical conductivity (EC) [23], a 1:10 combination of biochar and distilled water was made. To assess the nitrogen (N) content, the biochar samples were subjected to digestion and distilled, utilising the Kjeldahl distillation technique [24]. Using HNO_3_-HClO_4_, biochar sample as digested and then phosphate (P) and potassium (K) concentrations in the biochar were evaluated [25]. Then, utilizing a spectrophotometer and the ammonium vanadate-ammonium molybdate yellow color procedure, the phosphorus (P) content was determined. A flame photometer was used to measure the potassium (K^+^) content [26]. Table 1 lists the physicochemical features of biochar.

**Table 1:** Pre-experimental soil, biochar and irrigation water characteristics

Soil	Values	Biochar	Values	Irrigation	Values
pH	8.01	pH	7.11	pH	7.21
SOC (%)	0.60	EC*e* (dS/m)	3.39	EC (µS/cm)	301
TN (%)	0.030	Volatile Matter (%)	45	Carbonates (meq./L)	0.00
EP (mg/kg)	4.12	Fixed carbon (%)	55	Bicarbonates (meq./L)	4.19
AK (mg/kg)	107	TN (%)	0.05	Chloride (meq./L)	0.15
Sand (%)	25	TP (%)	0.01	Ca + Mg (meq./L)	3.95
Silt (%)	40	TK (%)	0.02	Sodium (mg/L)	171
Clay (%)	35	Surface area (m²/g)	450	TN = Total Nitrogen EP = Extractable Phosphorus AK = Available Potassium CEC = Cation Exchange Capacity	
Texture	Clay Loam	CEC (meq./100 g)	500		

### Carboxymethyl cellulose

Carboxymethyl cellulose (CMC) was procured from a certified local supplier of Sigma-Aldrich. The CMC product details are as follows: Product Number: PHR2726-2G, Lot Number: LRAD6430, Physical Form: Solid, Color: White.

### Treatments and experimental plan

**Fig. 1:**
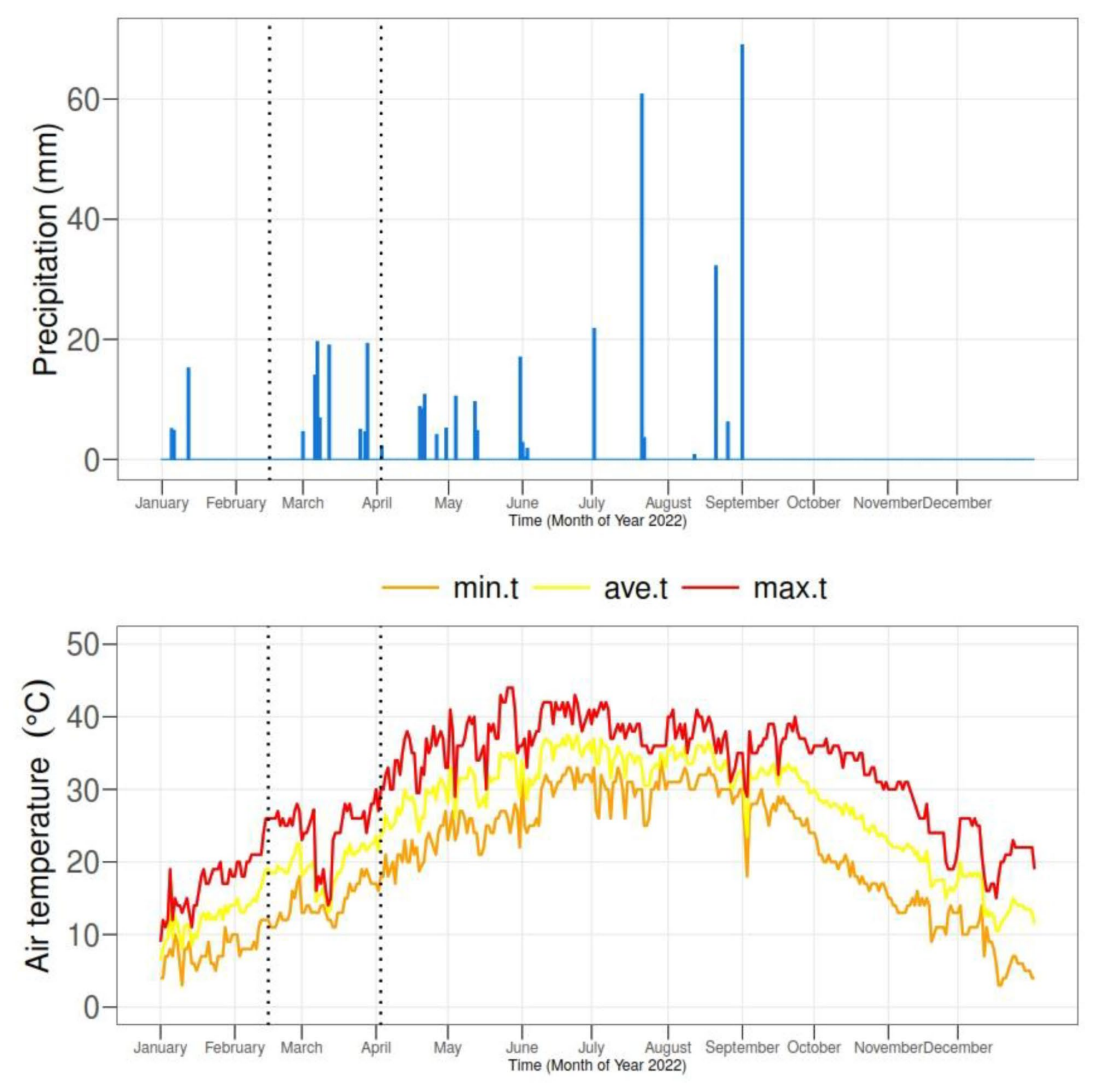
Climatic data of the experimental site

There were 3 levels of deashed biochar (DAB) applied in the soil. The DAB levels include 0, 0.5% and 1.0%. Two levels of CMC were applied as foliar, i.e., 0 and 25mM. All the treatments were applied on the maize plants under no drought stress (70%FC) and drought stress (40%FC) following a completely randomized design (CRD). The climatic data of the experimental site is provided in Fig. 1.

### Seeds collection and sterilization

The maize seeds (Cimmyt-Pak) utilized in the present research came from a licensed seed supplier who was approved by the Punjab government in Pakistan. Only strong, healthy seeds were chosen to verify the seeds’ integrity; broken and weak seeds were not included. The chosen seeds underwent a surface-sterilization procedure before being sown. To do this, the seeds were first treated with a 5% solution of sodium hypochlorite, followed by three washings with 95% ethanol. The seeds were then rinsed three times in sterilized deionized water to eliminate any remaining sterilizing chemicals [27].

### Seeds sowing and thinning

A total of 10 seeds were sown, in each pot containing 15 kg of soil. After germination, the number of seedlings in each pot was reduced to 2 through thinning.

### Drought

In order to investigate the effects of drought stress on maize plant physiology and growth, a controlled experiment was designed to establish two distinct soil moisture conditions: a no drought stress condition referred to as 70% field capacity (70FC) and drought stress (DS) condition denoted as 40% field capacity (40FC) [28].

### Data gathering and harvesting

Plants were collected for data collection after 50 days of sowing. Weights of fresh shoot and roots were measured soon after harvest. Samples were oven-dried at 65 °C for 72 h to get consistent weight for determining the dry mass of the shoot and roots.

### Chlorophyll contents and carotenoids

Arnon’s approach was used for the determination of chlorophyll a, chlorophyll b, and total chlorophyll in fresh maize leaves [29]. A mixture of 80% acetone was used for the extraction. For chlorophyll a and b, absorbance measurements were made at wavelengths of 663 nm and 645 nm, respectively.

$${\text{Chlorophyll}}\,{\text{a}}\left( {\frac{{{\text{mg}}}}{{\text{g}}}} \right) = \frac{{\left( {12.7 \times {\text{A}}663} \right) - \left( {2.69 \times {\text{A}}645} \right) \times {\text{V}}}}{{1000 \times {\text{W}}}}$$

$${\text{Chlorophyll}}\,{\text{b}}\left( {\frac{{{\text{mg}}}}{{\text{g}}}} \right) = \frac{{\left( {22.9 \times {\text{A}}645} \right) - \left( {4.68 \times {\text{A}}663} \right) \times {\text{V}}}}{{1000 \times {\text{W}}}}$$

$$\text{T}\text{o}\text{t}\text{a}\text{l} \text{C}\text{h}\text{l}\text{o}\text{r}\text{o}\text{p}\text{h}\text{y}\text{l}\text{l} \left(\frac{\text{m}\text{g}}{\text{g}}\right)= 20.2\left(\text{O}\text{D} 645\right)+8.02\left(\text{O}\text{D} 663\right)\times \text{V}/1000 \left(\text{W}\right)$$

### Gas exchange characteristics

The CI-340 Photosynthesis system by CID, Inc. USA was used as the infrared gas analyzer for determining the leaf’s stomatal conductivity, net rate of photosynthetic activity, and net transpiration rate, respectively. Within 10:30 and 11:30 a.m. on a bright day, while the light level was sufficient for photosynthesis, assessments were made [30].

### SOD

The inhibition of nitro blue tetrazolium (NBT) reduction was studied to ascertain SOD activity. The absorbance was taken at 560 nm. [31].

### POD

Observing the oxidation of an appropriate substrate, including guaiacol or o-dianisidine, was used to measure POD activity. At 420 nm wavelength, the rise in absorbance brought on by substrate oxidation was quantified [32].

### CAT

Catalase (CAT) activity was quantified by measuring the breakdown of hydrogen peroxide H_2_O_2_ and the subsequent reduction in absorbance at 240 nm, indicative of H_2_O_2_ decomposition.

### APX

The oxidation of ascorbate in the presence of H_2_O_2_ was observed for APX activity at 240 nm [33]. The oxidation of ascorbate in the presence of H_2_O_2_ was observed for APX activity.

### MDA

The MDA, was measured by forming a colored compound by reacting the sample extract with thiobarbituric acid (TBA). The complex’s absorption was determined at 532 nm.

### Electrolyte leakage

The leaves were first washed with water that was deionized to get rid of any exterior pollutants before the testing was done. Then, utilizing a steel cylinder with a 1 cm diameter, uniform-sized leaf pieces measuring around one gram were produced. The leaf fragments were next put into several tubes for testing with 20 ml of deionized water. To facilitate the passage of electrolytes from the leaf tissues into the water, the test tubes were left to incubate at 25 °C for 24 h. An EC meter that was already validated was used to test the water solution’s electrical properties (EC1) after the incubation time. The test tubes were then heated in a water bath for 20 min at 120 °C to measure the second electrical conductivity (EC2) (Lutts et al., 1996).

$${\rm{Electrolyte}}\,{\rm{leakage}}\,{\rm{(\% )}}\,{\rm{ = }}\,\left( {{{{\rm{EC1}}} \over {{\rm{EC2}}}}} \right)\, \times \,100$$

### Statistical analysis

Analysis of variance was applied to assess the collected data was statistically analyzed in R Software (Version) using a linear mixed model [34]. ANOVA values are provided in Tables 2 and 3. The means were compared using Tukey multiple comparison tests at *p* < 0.05. The figures were created using origin software.

**Table 2:** *P*-value of main and interaction effect of deashed biochar (DAB) and carboxymethyl cellulose (CMC) on shoot length, shoot fresh weight, shoot dry weight, root fresh weight, root dry weight, root length, number of leaves, leaf fresh weight, leaf dry weight, chlorophyll a, chlorophyll b, total chlorophyll, carotenoids and root potassium under no drought and drought stress

Treatments	Shoot length	Shoot fresh weight	Shoot dry weight	Root fresh weight	Root dry weight	Root length	Number of leaves	Leave fresh weight	Leave dry weight	Chlorophyll a	Chlorophyll b	Total chlorophyll	Carotenoids	Root potassium
	No Drought Stress													
DAB	0.01	< 0.01	0.01	0.04	0.04	0.11	0.21	0.13	0.11	0.11	0.02	0.08	0.10	0.02
CMC	< 0.01	< 0.01	< 0.01	< 0.01	< 0.01	< 0.01	< 0.01	< 0.01	< 0.01	< 0.01	< 0.01	< 0.01	< 0.01	< 0.01
DAB×CMC	0.62	0.33	0.40	0.26	0.82	0.97	0.65	0.76	0.87	0.81	0.63	0.46	0.67	0.51
	Drought Stress													
DAB	< 0.01	< 0.01	< 0.01	< 0.01	< 0.01	< 0.01	< 0.01	< 0.01	< 0.01	< 0.01	< 0.01	< 0.01	< 0.01	< 0.01
CMC	< 0.01	< 0.01	< 0.01	< 0.01	< 0.01	< 0.01	< 0.01	< 0.01	< 0.01	< 0.01	< 0.01	< 0.01	< 0.01	< 0.01
DAB×CMC	0.25	0.27	0.01	0.01	0.12	0.21	0.36	0.36	0.01	0.01	0.19	0.01	0.02	0.522

**Table 3:** *P*-value of main and interaction effect of deashed biochar (DAB) and carboxymethyl cellulose (CMC) on photosynthetic rate, transpiration rate,, stomatal conductance, leaves nitrogen, leaves phosphorus, leaves potassium, root nitrogen, electrolyte leakage, POD, SOD, catalase, ascorbic acid, H_2_O_2_, MDA and root phosphorus under no drought and drought stress

Treatments	Photosynthetic rate	Transpiration rate	Stomatal conductance	Leave Nitrogen	Leave Phosphorus	Leave Potassium	Root Nitrogen	Electrical Leakage	POD	SOD	Catalase	Ascorbic acid	H_2_O_2_	MDA	Root Phosphorus
		No Drought Stress													
DAB	0.10	0.18	0.06	0.016	0.01	0.09	0.02	< 0.02	0.09	0.12	0.01	0.01	0.08	0.02	0.11
CMC	< 0.01	< 0.01	< 0.01	< 0.01	< 0.01	< 0.01	< 0.01	< 0.01	< 0.01	< 0.01	< 0.01	< 0.01	< 0.01	< 0.01	< 0.01
DAB×CMC	0.85	0.99	0.76	0.76	0.60	0.51	0.02	0.92	0.48	0.284	0.49	0.08	0.93	0.95	1.00
		Drought Stress													
DAB	< 0.001	< 0.001	< 0.001	< 0.001	< 0.001	< 0.001	< 0.001	< 0.001	< 0.001	< 0.001	< 0.001	< 0.001	< 0.001	< 0.001	< 0.001
CMC	< 0.001	< 0.001	< 0.001	< 0.001	< 0.001	< 0.001	< 0.001	< 0.001	< 0.001	< 0.001	< 0.001	< 0.001	< 0.001	< 0.001	< 0.001
DAB×CMC	0.54	< 0.001	0.041	1.00	0.04	0.017	< 0.001	< 0.001	< 0.001	0.587	0.587	0.539	0.199	0.11	0.02

## Results

### Shoot length, shoot fresh and dry weight

In the absence of both DAB and CMC (70 FC, 0 DAB + 0 CMC), the mean shoot length was 38.09 cm. However, when CMC was added, there was a noticeable percentage increase of 16.96% in shoot length over the control (0 DAB + 0 CMC) under 70 FC. In contrast, the application of 0.5 DAB resulted in a 9.11% increase in shoot length from the control (0 DAB + 0 CMC) under no stress. When both 0.5 DAB and 25 CMC were applied, a significant 28.43% increase was observed in the shoot length over the control under no drought stress (70 FC). Finally, under 70 FC conditions, treatment 1 DAB showed 5.17% increase in the shoot length as compared to the control. When 1 DAB was combined with 25CMC there was a remarkable 24.36% increase in shoot length related to the control under no drought stress (70 FC). Under drought stress conditions with no DAB and CMC, the mean shoot length was 26.90 cm. However, when 25 CMC treatment was added under drought stress, there was a significant 20.04% increase in the shoot length observed over the control under drought stress. Similarly, the application of treatment 0.5 DAB under drought stress led to a 6.32% increase in the shoot length from the control. When both 0.5 DAB and 25 CMC treatments were combined, 26.17% increase in shoot length was recorded parallel to the control under drought stress. Moreover, 1 DAB treatment under drought stress conditions showed 13.46% rise in the shoot length with respect to the control. When 1 DAB was combined with 25 CMC, there was a remarkable 32.23% increase, resulting in the shoot length related to the control in drought stress.

The average shoot fresh weight was 170.92 g/plant when DAB and CMC under 0 DAB and 0 CMC in 70 Fc condition. Under 70 FC, in comparison to the control group (0 DAB + 0 CMC), the application of 25 CMC resulted in a significant 5.63% increase in shoot fresh weight. Similarly, the use of 0.5 DAB led to a 3.81% increase, while the combination of 0.5 DAB and 25 CMC showed a substantial 12.94% increase in shoot fresh weight parallel to the control under 70 FC. However, when 1 DAB treatment was applied, there was only a modest 2.40% increase in shoot fresh weight compared to the control in the 70 FC condition. The most significant increase was observed when 1 DAB was combined with 25 CMC, resulting in a notable 10.41% rise in shoot fresh weight compared to the control under no drought stress (70 FC). In comparison to the drought stress control group (0 DAB + 0 CMC), the application of 25 CMC led to a remarkable 14.21% increase in shoot fresh weight. Additionally, 0.5 DAB treatment resulted in a 4.50% increase, while the combination of 0.5 DAB and 25 CMC showed a substantial 18.94% increase in shoot fresh weight over the control under drought stress. Furthermore, when 1 DAB was applied during drought stress, there was a notable 9.33% increase in shoot fresh weight compared to the control. The most significant increase was observed with the combination of 1 DAB and 25 CMC, which resulted in a remarkable 24.53% rise in shoot fresh weight contrasted to the control under drought stress conditions.

In a 70 FC circumstances, the control group with no DAB and CMC (0 DAB + 0 CMC) had a mean shoot dry weight of 17.25 g/plant. When 25 CMC was introduced, there was a noTable 14.49% increase in shoot dry weight linked to the control under no stress (70 FC). Similarly, the application of 0.5 DAB resulted in a 10.43% increase in shoot dry weight over the control under the same 70 FC conditions. Combining 0.5 DAB with 25 CMC treatment under no drought stress (70 FC), led to a substantial 24.23% increase in shoot dry weight contrasted to the control. The 1 DAB treatment showed a significant 4.00% increase in shoot dry weight, and when 1 DAB was combined with 25 CMC, there was a noTable 22.32% increase relative to the control under 70FC conditions. Under drought stress conditions, the control group (0 DAB + 0 CMC) had a mean shoot dry weight of 11.93 g/plant. The addition of 25 CMC during drought stress resulted in a significant 24.98% increase in shoot dry weight compared to the control. In comparison to the baseline treatment, the application of 0.5 DAB treatment led to a 12.15% increase in shoot dry weight under drought stress. When 0.5 DAB was combined with 25 CMC during drought stress, a remarkable 31.10% increase in shoot dry weight was observed compared to the control. The 1 DAB treatment exhibited a 22.05% increase, and when 1 DAB was combined with 25 CMC, there was a substantial 38.47% increase in shoot dry weight relative to the control during drought stress (Table 4).

**Table 4:** The effect of carboxymethyl cellulose (CMC) and deashed biochar (DAB) on shoot and root length, shoot and root fresh and dry weights of maize cultivated under no drought and drought stress

DAB (%)	Shoot Length (cm)		Shoot Fresh Weight (g)			Shoot Dry Weight (g)		
	0 CMC	25 CMC	0 CMC	25 CMC	0 CMC		25 CMC	
	Field Capacity 70							
0	38.09 ± 0.67a	44.55 ± 1.21c	170.92 ± 1.76a	180.55 ± 1.79 c	17.25 ± 0.16a		19.75 ± 0.46b	
0.5	41.56 ± 0.95b	48.92 ± 0.13d	177.44 ± 0.97bc	193.04 ± 1.75e	19.05 ± 0.14b		21.43 ± 0.17c	
1.0	40.06 ± 0.49b	47.37 ± 0.64d	175.02 ± 1.32b	188.71 ± 1.93d	17.94 ± 0.59a		21.1 ± 0.16c	
	Drought Stress							
0	28.6 ± 0.24b	33.94 ± 0.53e	134.8 ± 1.25b	153.43 ± 2.19e	13.38 ± 0.59b		15.64 ± 0.47d	
0.5	30.52 ± 0.65c	35.57 ± 0.95f	141.04 ± 1.05c	160.64 ± 3.08f	14.56 ± 0.18c		16.52 ± 0.3e	
1.0	26.9 ± 0.67a	32.29 ± 0.65d	129 ± 3.39a	147.33 ± 2.75d	11.93 ± 0.7a		14.91 ± 0.08c	
DAB (%)	Root Length (cm)		Root Fresh Weight (g)			Root Dry weight (g)		
	Field Capacity 70							
0	18.72 ± 0.21a	21.72 ± 0.46bc	25.71 ± 0.29a	30.04 ± 0.33c	4.54 ± 0.06a		6.12 ± 0.21c	
0.5	20.77 ± 0.40b	23.45 ± 0.80d	28.39 ± 0.26b	34.22 ± 0.34e	5.25 ± 0.17b		6.72 ± 0.19d	
1.0	19.52 ± 0.39a	22.49 ± 0.24 cd	26.51 ± 0.31a	32.64 ± 1.24d	4.91 ± 0.02b		6.42 ± 0.05 cd	
	Drought Stress							
0	11.33 ± 0.22b	16.11 ± 0.31e	20.60 ± 0.17a	22.30 ± 0.28d	2.24 ± 0.15a		3.33 ± 0.10d	
0.5	13.22 ± 0.64c	17.54 ± 0.43f	21.18 ± 0.13b	23.68 ± 0.54e	2.54 ± 0.18b		3.98 ± 0.04e	
1.0	10.33 ± 0.24a	14.99 ± 0.56d	21.72 ± 0.25c	24.52 ± 0.29f	2.94 ± 0.06c		4.20 ± 0.16f	
DAB (%)	Number of leaves		Leave Fresh weight (g)			Leave Dry weight (g)		
	Field Capacity 70							
0	8.16 ± 0.07a	9.3 ± 0.11c	41.08 ± 1.27a	47.36 ± 1.2c	8.14 ± 0.18a		10.57 ± 0.18c	
0.5	8.77 ± 0.11b	9.79 ± 0.2d	44.81 ± 0.54b	54.38 ± 0.74 e	9.82 ± 0.57b		11.63 ± 0.20d	
1.0	8.28 ± 0.04a	9.54 ± 0.04 cd	42.81 ± 0.39ab	49.9 ± 0.61d	8.77 ± 0.14 a		11.08 ± 0.16 cd	
	Drought Stress							
0	28.6 ± 0.24b	33.94 ± 0.53e	134.8 ± 1.25b	153.43 ± 2.19e	13.38 ± 0.59b		15.64 ± 0.47d	
0.5	30.52 ± 0.65c	35.57 ± 0.95f	141.04 ± 1.05c	160.64 ± 3.08f	14.56 ± 0.18c		16.52 ± 0.3e	
1.0	26.9 ± 0.67a	32.29 ± 0.65d	129 ± 3.39a	147.33 ± 2.75d	11.93 ± 0.7a		14.91 ± 0.08c	

Values are mean ± standard deviation (*n* = 3)

### Root fresh weight

Under 70FC (no drought stress), the control group (0 DAB + 0 CMC) exhibited a mean root fresh weight of 25.71 g/plant. When 25 CMC was introduced under the 70 FC conditions, there was a significant 16.84% increase and the application of 0.5 DAB, indicating a 10.42% increase in root fresh weight compared to the control. The combination of 0.5DAB and 25 CMC resulted in a remarkable 33.10% increase in root fresh weight, contrasting to the control (70 FC). In contrast, when 1 DAB was applied under no stress (70 FC), representing a modest 3.11% increase in root fresh weight over the control. However, combining 1 DAB with 25 CMC as compared to the control led to a more substantial 26.95% increase in root fresh weight under 70FC conditions. During drought stress conditions, the control group (0 DAB + 0 CMC) exhibited a mean root fresh weight of 20.60 g/plant. The introduction of 25 CMC under drought stress resulted in an 8.25% increase, and treatment 0.5 DAB showed a slight 2.82% increase in root fresh weight assessed to the control. The combination of 0.5 DAB and 25 CMC yielded a 14.95% increase in root fresh weight than the control under drought stress. Similarly, using 1DAB during drought stress conditions represented a 5.44% increase in root fresh weight over the control. When 1 DAB was combined with 25 CMC during drought stress, there was a substantial 19.03% increase in root fresh weight from the control.

### Root dry weight

The root dry weight was measured 4.54 g/plant under no drought stress (40 FC) with no DAB and CMC (0 DAB + 0 CMC). When 25 CMC was introduced under 70 FC conditions, there was a notable 34.80% increase in root dry weight over the control. Applying 0.5 DAB treatment resulted in a 15.64% increase in root dry weight over the control (0 DAB + 0 CMC). Combining 0.5 DAB with 25 CMC led to a substantial 48.02% increase, and using 1DAB treatment in the 70 FC condition reflected an 8.15% increase in root dry weight matched to the control. Furthermore, when 1 DAB was combined with 25 CMC under 70 FC conditions, a significant 41.41% increase in root dry weight was observed over the control under no stress (70 FC). Under drought stress conditions with no DAB and CMC (0DAB + 0 CMC), the mean root dry weight was 2.24 g/plant. However, when 25 CMC treatment was applied during drought stress, there was a substantial 48.66% increase in root dry weight from the control group. Using 0.5 DAB treatment during drought stress represents a 13.39% increase in root dry weight over the control. Combining 0.5 DAB with 25 CMC during drought stress induced a remarkable 77.68% increase in root dry weight in comparison to the control. Employing 1 DAB treatment during drought stress conditions resulted in a 31.25% increase in root dry weight associated to the control. Furthermore, when 1 DAB was combined with 25 CMC during drought stress, a significant 87.50% increase in root dry weight was observed related to the control.

### Root length, number of leaves, leaves fresh and dry weights

The control group (0 DAB + 0 CMC) exhibited an average root length of 18.72 cm under 70 FC conditions. When 25 CMC treatment was introduced to the plant, there was a notable 16.03% increase in root length over the control treatment under no drought stress (70 FC). Similarly, the application of 0.5 DAB resulted in a 10.95% increase in the root length evaluated to the control under no drought stress (70 FC). The combined treatment of 0.5 DAB and 25 CMC showed a substantial 25.27% increase in root length under no drought stress (70 FC) over the control. On the other hand, the 1DAB treatment yielded a modest 4.27% increase in root length related to the control. However, when 1 DAB was combined with 25 CMC, a more significant 20.14% increase in root length was observed under 70 FC over the control. In contrast, under drought stress conditions, the control group (0 DAB + 0 CMC) had an average root length of 10.33 cm. The introduction of 25 CMC led to a remarkable 45.11% increase in root length under drought stress over the control. Similarly, the application of 0.5 DAB represents a 9.68% increase in root length linked to the control. The combined treatment of 0.5 DAB and 25 CMC showed a substantial 55.95% increase in root length, and on the other hand, the 1 DAB treatment corresponded to a notable 27.98% increase in root length from the control under no drought stress. When 1 DAB was combined with 25 CMC during drought stress, a remarkable 69.80% increase in root length was observed, contrasting with the control.

Under 70 F C conditions, the application of 25 CMC led to a notable 13.97% increase in the number of leaves compared to the control (0 DAB + 0 CMC). Similarly, when 0.5DAB was applied under 70 FC, there was a 7.48% increase in the number of leaves in comparison to the control. Combining 0.5 DAB with 25 CMC resulted in a significant 19.98% increase in the number of leaves contrasted to the control under 70 FC. On the other hand, the use of 1 DAB showed a modest 1.47% increase in the number of leaves competed to the control under 70 FC conditions. When 1 DAB was combined with 25 CMC, a 16.91% increase in the number of leaves was observed relative to the control under the 70 FC stress conditions. Under drought stress conditions, the control group of the leaves number was recorded 5.12. The application of 25 CMC during drought stress resulted in a substantial 33.98% increase in the number of leaves evaluated to the control. Likewise, the control using 0.5 DAB treatment led to an 8.98% increase in the number of leaves during drought stress. Combining 0.5DAB with 25 CMC caused a remarkable 43.16% increase in the number of leaves equaled to the control under drought stress conditions. Furthermore, the application of 1 DAB showed a 21.29% increase in the number of leaves matched to the control under drought stress. When 1 DAB was combined with 25 CMC during drought stress, there was a striking 53.13% increase in the number of leaves relative to the control.

Under no drought stress (70 FC) with no DAB and CMC (0 DAB + 0 CMC), the mean leaves fresh weight was 41.08 g/plant. When 25 CMC was introduced under the 70FC conditions, there was a notable 15.29% increase in leaves fresh weight compared to the control. Similarly, applying 0.5 DAB resulted in a 9.08% increase in leaves fresh weight relative to the control, while combining 0.5 DAB with 25 CMC led to a substantial 32.38% increase under no drought stress (70 FC). The use of 1 DAB under 70 FC conditions resulted in a significant 4.21% increase in leaves fresh weight related to the control, and when 1 DAB was combined with 25 CMC, a 21.47% increase was observed. Under drought stress conditions, without DAB and CMC (0 DAB + 0 CMC), the mean leaves fresh weight was 26.27 g/plant. However, the application of 25 CMC during drought stress induced a significant 33.35% increase in leaves fresh weight associated to the control under the drought stress conditions. Using 0.5 DAB during drought stress led to a 14.24% increase in leaves fresh weight related to the control, and when 0.5 DAB was combined with 25 CMC, a substantial 40.20% increase in fresh weight was recorded relative to the control. Similarly, the use of 1 DAB during drought stress conditions resulted in a 22.23% increase in fresh weight equated to the control, and when 1 DAB was combined with 25 CMC, a remarkable 47.16% increase in fresh weight was observed related to the control.

The mean leaf dry weight for the control group (0 DAB + 0 CMC) at 70 FC conditions was 8.14 g/plant. However, when 25 CMC treatment was introduced to the plant, there was a significant 29.85% increase in leaves dry weight under no drought stress (70 FC) over the control group. Similarly, under no drought stress (70 FC), the application of 0.5DAB resulted in a 20.64% increase in dry weight linked to the control. Combining 0.5DAB with 25 CMC treatment exhibited the most substantial 42.87% boost in dry weight over the control under no drought stress (70 FC). On the other hand, 1 DAB treatment showed a modest 7.74% increase in dry weight, and when combined with 25 CMC, it yielded a 36.12% increase in dry weight under 70 FC conditions in comparison to the control treatment. Under drought stress conditions, the control group (0 DAB + 0 CMC) had a mean dry weight of 5.11 g/plant. The introduction of 25 CMC during drought stress resulted in a 29.35% increase in dry weight over the control. Similarly, 0.5 DAB treatment showed a 6.46% increase in dry weight related to the control under drought stress. The combination of 0.5 DAB with 25 CMC treatment compared to the control demonstrated a substantial 40.12% increase in dry weight. In contrast to the control, 1 DAB treatment displayed a 13.11% increase in dry weight, and when combined with 25 CMC during drought stress, it exhibited a remarkable 50.29% increase in dry weight under drought stress (Table 5).

**Table 5:** The effect of carboxymethyl cellulose (CMC) and deashed biochar (DAB) on chlorophyll a, b, total, carotenoids and electrolyte leakage of maize cultivated under no drought and drought stress

DAB (%)	Chlorophyll a (mg g^− 1^)		Chlorophyll b (mg g^− 1^)		Total Chlorophyll (mg g^− 1^)		Carotenoids (mg g^− 1^)			
	0 CMC	25 CMC	0 CMC	0 CMC	0 CMC	25 CMC	0 CMC	25 CMC		
	Field Capacity 70									
0	1.23 ± 0.03a	1.23 ± 0.03a	0.34 ± 0.01a	0.36 ± 0.01c	1.48 ± 0.03a	1.68 ± 0.02d	0.76 ± 0.01a	0.83 ± 0.01c		
0.5	1.39 ± 0.02 cd	1.39 ± 0.02 cd	0.36 ± 0.01bc	0.38 ± 0.01d	1.61 ± 0.04c	1.79 ± 0.02e	0.81 ± 0.01b	0.88 ± 0.01d		
1.0	1.23 ± 0.03a	1.23 ± 0.03a	0.34 ± 0.01ab	0.38 ± 0.01d	1.56 ± 0.02b	1.71 ± 0.01d	0.77 ± 0.01a	0.86 ± 0.01d		
	Drought Stress									
0	0.77 ± 0.01a	0.96 ± 0.02d	0.26 ± 0.01a	0.3 ± 0.01d	1.08 ± 0.03a	1.29 ± 0.03d	0.31 ± 0.03a	0.52 ± 0.04d		
0.5	0.81 ± 0.02b	1.01 ± 0.02e	0.27 ± 0.01b	0.31 ± 0.01e	1.16 ± 0.03b	1.34 ± 0.02e	0.39 ± 0.01b	0.6 ± 0.04e		
1.0	0.91 ± 0.02c	1.08 ± 0.05f	0.28 ± 0.01c	0.33 ± 0.01f	1.23 ± 0.03c	1.41 ± 0.04f	0.44 ± 0.03c	0.68 ± 0.01f		
DAB (%)	Photosynthetic rate (µmol CO_2_/m^2^/s)		Transpiration rate (mmol H_2_O/m^2^/s)		Stomatal Conductance (mol H_2_O/m^2^/S)		Electrolyte Leakage (%)			
	Field Capacity 70									
0	18.87 ± 0.16a	21.72 ± 0.67c	1.27 ± 0.03a	1.55 ± 0.04d	2.05 ± 0.02a	2.32 ± 0.02d	40.27 ± 0.99c	34.26 ± 0.54e		
0.5	20.23 ± 0.27b	22.77 ± 0.11d	1.46 ± 0.01c	1.72 ± 0.02f	2.24 ± 0.03c	2.47 ± 0.03f	35.48 ± 0.22a	26.9 ± 0.75 cd		
1.0	19.34 ± 0.19a	22.31 ± 0.14 cd	1.38 ± 0.03b	1.65 ± 0.03e	2.14 ± 0.02b	2.38 ± 0.02e	37.05 ± 0.91b	30.74 ± 1.47d		
	Drought Stress									
0	15.22 ± 0.13a	17.21 ± 0.48d	0.92 ± 0.03a	1.12 ± 0.01d	1.27 ± 0.03a	1.63 ± 0.07d	58.93 ± 0.27b	50.8 ± 2.70d		
0.5	15.74 ± 0.26b	17.86 ± 0.1e	1 ± 0.03b	1.15 ± 0.02d	1.4 ± 0.06b	1.75 ± 0.03e	57.78 ± 0.56a	44.03 ± 0.5d		
1.0	16.38 ± 0.18c	18.47 ± 0.21f	1.08 ± 0.03c	1.22 ± 0.02e	1.52 ± 0.04c	1.93 ± 0.06f	55.46 ± 1.77a	42.6 ± 0.62c		

Values are mean ± standard deviation (*n* = 3)

### Chlorophyll a, b, total chlorophyll and carotenoids

In 70 FC conditions, the control group (0 DAB + 0 CMC) exhibited chlorophyll a level of 1.18 mg/g. There was a substantial 14.41% increase in chlorophyll a content from the control when treatment 25 CMC was applied to the plant in the 70 FC (no drought stress). Like the control group, the administration of 0.5DAB in the absence of stress (70FC) increased the chlorophyll a content by 11.02%. Combining 0.5 DAB with 25 CMC treatment in the 70FC condition resulted in a remarkable 22.03% increase in chlorophyll a content related to the control. The use of 1 DAB under 70 FC conditions yielded a modest 4.24% increase in chlorophyll a content from the control. However, when 1 DAB was combined with 25 CMC in the same conditions, there was a significant 17.80% increase in chlorophyll a content contrasting to the control. In contrast, under drought stress conditions, the control group (0 DAB + 0 CMC) displayed chlorophyll a content of 0.77 mg/g. The introduction of 25 CMC during drought stress resulted in a substantial 24.68% increase in chlorophyll a content from the control. Applying 0.5 DAB during drought stress caused a moderate 5.19% increase in chlorophyll a content in comparison to the control. Combining 0.5 DAB with 25 CMC contrasting with the control under drought stress conditions led to a significant 31.17% increase in chlorophyll a content. Similarly, the use of 1 DAB during drought stress conditions produced an 18.18% increase, and treatment 1 DAB was combined with 25 CMC in the drought stress conditions, there was a remarkable 40.26% increase in the chlorophyll a content in contrast to the control.

The chlorophyll b content of the control (0 DAB + 0 CMC) group was measured to be 0.34 mg/g in a non-stressful condition (70 FC). When 25 CMC was applied under 70FC conditions, there was a 5.88% increase in chlorophyll b content from the control, and the application of 0.5 DAB resulted in the same 5.88% increase. However, the combination of 0.5 DAB and 25 CMC led to a more substantial increase of 11.76% in chlorophyll b content estimated to the control under no drought stress (70 FC). On the other hand, the 1 DAB treatment showed no change in chlorophyll b content relative to the control under 7 0FC conditions, and when 1 DAB was combined with 25 CMC, there was an 11.76% increase in chlorophyll b content. Under drought stress conditions, the control group (0 DAB + 0 CMC) exhibited a reduced chlorophyll b content of 0.26 mg/g. The introduction of 25 CMC during drought stress resulted in a notable 15.38% increase in chlorophyll b content compared to the control, and with the 0.5 DAB treatment 3.85% increase was recorded. When both 0.5 DAB and 25 CMC treatments were combined during drought stress, a significant 19.23% increase in chlorophyll b content was recorded. The 1 DAB treatment also demonstrated an increase of 7.69% in chlorophyll b content contrasted to the control under drought stress. Notably, when 1 DAB was combined with 25 CMC during drought stress, there was a substantial 26.92% increase in chlorophyll b content relative to the control.

Under 70 FC conditions, the control group (0 DAB + 0 CMC) exhibited a mean total chlorophyll content of 1.48 mg/g. There was a 13.51% increase in total chlorophyll content when 25 CMC was added in comparison with the control over 70 FC. In addition, treatment with 0.5 DAB led to a rise in total chlorophyll content of 8.78%, while treatment with 0.5 DAB and 25 CMC together yielded a substantial boost in total chlorophyll content of 20.95% as compared to the control under 70 FC. Under 70 FC scenarios, the use of 1 DAB induced a 5.41% rise in total chlorophyll, while the combination of 1 DAB plus 25 CMC treatment yielded a 15.54% boost in total chlorophyll content in comparison to the control. Under drought stress conditions, the control group (0 DAB + 0 CMC) had a mean total chlorophyll content of 1.08 mg/g. With the introduction of 25 CMC, there was a notable 19.44% increase in total chlorophyll content, and the application of 0.5 DAB during drought stress led to a 7.41% increase in total chlorophyll content from the control. The usage of 1DAB treatment under drought stress scenarios resulted in a 13.89% rise in total chlorophyll content, while the combination treatment of 0.5 DAB and 25 CMC produced a significant 24.07% increase in total chlorophyll content over the control. When 1 DAB was combined with 25 CMC, there was a remarkable 30.56% increase in total chlorophyll content under drought stress from the control.

In the 70 FC condition, the mean carotenoid content was 0.76 mg/g for the control group (0 DAB + 0 CMC). When 25 CMC was introduced, there was a 9.21% increase in carotenoid content, and the application of 0.5 DAB led to a 6.58% increase in carotenoid content over the control under no drought stress (70 FC). while combining 0.5 DAB with 25 CMC resulted in a substantial 15.79% increase, and 1 DAB treatment showed a slight 1.32% increase in carotenoid content over the control under 70 FC. When combined with 1DAB + 25CMC treatment, a 13.16% increase was observed over the control under no stress (70 FC). In contrast, under drought stress conditions, the control group (0 DAB + 0 CMC) exhibited a mean carotenoid content of 0.31 mg/g. The introduction of 25 CMC during drought stress resulted in a remarkable 67.74% increase in carotenoid content and the application of 0.5 DAB treatment under drought stress conditions led to a significant 25.81% increase from the control. Combining 0.5 DAB with 25 CMC during drought stress induced a substantial 93.55% increase in carotenoid content than the control. The 1 DAB treatment during drought stress showed a noTable 41.94% increase, and when combined with 25 CMC, a remarkable 119.35% increase was observed over the control (Table 5).

### Photosynthetic rate, transpiration rate, stomatal conductance, and leave nitrogen

The photosynthetic rates of the control group were examined at 18.87 µmol CO_2_/m²/s under 70 FC with no DAB and 0 CMC. When 25 CMC treatment was applied, there was a notable 15.10% in photosynthetic rates increase over the control under 70 FC. Similarly, the application of 0.5 DAB resulted in a 7.21% increase and 0.5 DAB with 25 CMC showed a substantial 20.67% increase in photosynthetic rates in contrast to the control under no stress (70 FC). For 1 DAB treatment, there was a modest 2.49% increase, and treatment 1DAB was combined with 25 CMC, resulting in a notable 18.23% increase observed over the control in photosynthetic rates under 70 FC. Under drought stress, the control group (0 DAB + 0 CMC) exhibited a photosynthetic rate of 15.22 µmol CO2/m²/s. The introduction of 25 CMC resulted in a significant 13.07% increase and the application of 0.5 DAB showed a minor 3.42% increase in photosynthetic rates than the control under drought stress. Combining 0.5 DAB with 25 CMC demonstrated a substantial 17.35% increase and the use of 1 DAB treatment during drought stress led to a 7.62% increase in photosynthetic rates over the control. Remarkably, when 1 DAB was combined with 25 CMC, there was a substantial 21.35% increase in photosynthetic rates compared to the control under drought-stress conditions.

In the 70FC (non-stress) environment, the mean transpiration rate for the control group (0 DAB + 0 CMC) was 1.27 mmol H_2_O/m^2^/s. When 25 CMC treatment was applied, there was a notable 22.05% increase in the mean transpiration rate related to the control under 40FC. Similarly, the application of 0.5 DAB treatment resulted in a 14.96% increase in transpiration rate, while combining 0.5 DAB with 25 CMC led to a substantial 35.43% increase over the control under 70FC. Additionally, 1 DAB treatment under drought stress showed an 8.66% rise in transpiration rate, and when combined 1 DAB with 25 CMC, showed a 29.92% raised in comparison to the control under no drought stress (70 FC) conditions. Under drought stress conditions, the control group (0 DAB + 0 CMC) exhibited a mean transpiration rate of 0.92 mmol H_2_O/m^2^/s. When 25CMC was applied during drought stress, there was a significant 21.74% increase in transpiration rate compared to the control. The use of 0.5 DAB during drought stress resulted in an 8.70% increase in transpiration rate, and when combined with 25 CMC, there was a 25.00% increase in transpiration rate from the control. Furthermore, 1 DAB treatment during drought stress showed a 17.39% increase in transpiration rate, while the combination of 1 DAB with 25 CMC resulted in a remarkable 32.61% increase in transpiration rate equated to the control under drought stress conditions.

Stomatal conductance in the control group with no CMC and no DAB was recorded to be 2.05 mol H_2_O/m^2^/s under 70 FC. With the application of treatment 0.5 DAB + 25 CMC, representing a percentage increase of 20.49% over the control. Particularly, the addition of 25 CMC alone resulted in a 13.17% increase in stomatal conductance compared to the control, whereas 0.5 DAB contributed to a 9.27% increase under no drought stress (70 FC). The use of 1 DAB and 1 DAB + 25 CMC treatments demonstrated more modest increases of 4.39% and 16.10%, respectively, relative to the control under 70FC conditions. In contrast, under drought stress conditions, the control group showed an average stomatal conductance of was1.27 H_2_O/m^2^/s without CMC and DAB. The addition of 25 CMC during drought stress led to a substantial 28.35% increase in stomatal conductance contrasted to the control. Likewise, the application of 0.5 DAB and 0.5 DAB + 25 CMC treatments under drought stress resulted in noticeable increases of 10.24% and 37.80%, respectively, over the control. The use of 1 DAB treatment showed a 19.69% increase, while the combination of 1 DAB + 25 CMC treatment recorded a remarkable 51.97% increase in stomatal conductance relative to the control under drought stress conditions.

In the absence of both DAB and CMC (0 DAB + 0 CMC) in the 70 FC contexts, the baseline leaf N% measured 0.15%. Introducing 25CMC treatment under 70 FC conditions resulted in a noticeable 13.33% increase in leaf N% over the control. Similarly, applying treatment 0.5DAB, resulted in a 6.67% increase, and the combination of 0.5 DAB and 25 CMC led to a 13.33% increase in leaf N% above the control under no drought stress (70FC). In addition, using 1 DAB under 70 FC circumstances increased leaf N% by 6.67%, whereas combining 1 DAB with 25 CMC resulted in a significant 13.33% rise in leaf N%. Under drought stress conditions, the baseline leaf N% without any treatments (0 DAB + 0 CMC) was 0.13%. However, adding 25 CMC during drought stress induced a 7.69% increase in leaf N% in contrast to the control. Furthermore, applying 0.5 DAB alone or in combination with 25 CMC resulted in a 7.69% and 15.38% increase in leaf N%, respectively over the control under drought stress. Additionally, the use of 1 DAB during drought stress conditions led to a 7.69% increase in leaf N%, while combining 1 DAB with 25 CMC showed a substantial 15.38% increase in leaf N% related to the control treatment (Table 5).

### Leaves phosphorus and potassium

**Fig. 2:**
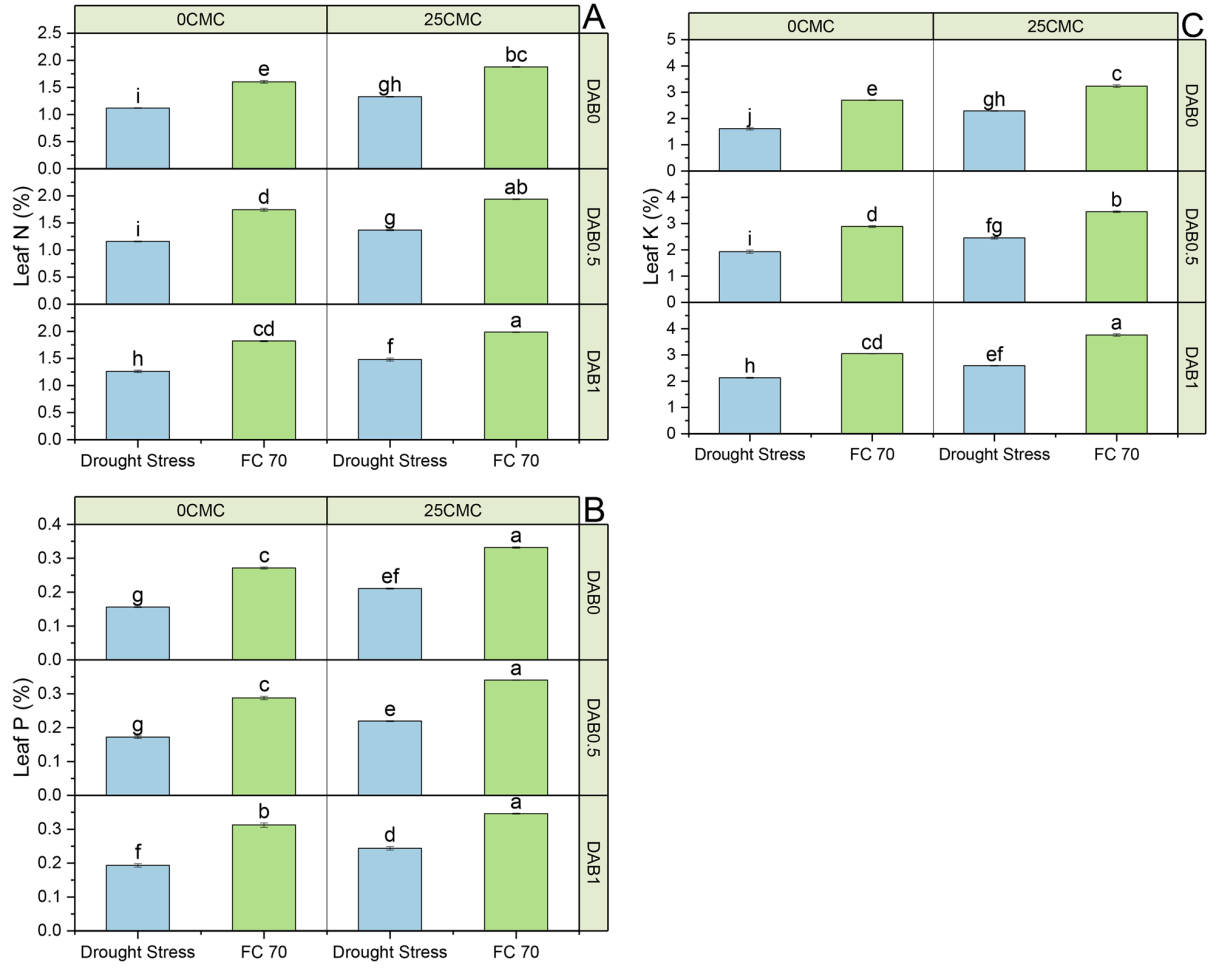
The effect of carboxymethyl cellulose (CMC) and deashed biochar (DAB) leaf nitrogen **(A)**, phosphorus **(B)** and potassium **(C)** of maize cultivated under no drought and drought stress. Dots on lines are mean of *n* = 3

In no drought stress (70 FC), the control group (0 DAB + 0 CMC) showed a leaf P content of 0.15%. When treatment 25 CMC was applied, there was a modest increase of 6.67% in leaf P content over the control group under 70 FC (Fig. 2). Conversely, the application of 0.5 DAB no change in leaf phosphorus content under 70 FC. However, when both 0.5 DAB and 25 CMC were combined, there was a notable 13.33% increase in leaf P content equaled to the control under no drought stress (70 FC). The highest increase was observed when 1 DAB was applied, resulting in a substantial 20.00% rise, and the combination of 1 DAB plus 25 CMC showed a 13.33% increase in leaf P content than the control under no drought stress (70 FC). The control group (0 DAB + 0 CMC) exhibited a leaf P content of 0.10%, which increased by 10.00% when 25 CMC was added. The application of 0.5 DAB showed no significant changes over the control under drought stress. However, when 0.5 DAB was combined with 25 CMC under drought stress, there was a substantial 20.00% increase in leaf P content relative to the control. The application of 1 DAB resulted in no significant changes or in combination with 25 CMC also resulted in a 20.00% increase in leaf P content compared to the control under drought stress conditions.

Under 70FC stress, the control group (0 DAB + 0 CMC) exhibited a leaf K value of 0.78 (%), while the introduction of 25 CMC treatment led to a 38.46% increase in comparison to the control under no drought stress (70 FC) (Fig. 1). When 0.5 DAB treatment was applied, there was a 29.49% increase in leaf K content, and the combination of 0.5 DAB with 25 CMC resulted in a substantial 53.85% increase in leaf K content over the control under 70 FC. Furthermore, 1 DAB treatment showed a 14.10% increase and when 1DAB was combined with 25 CMC, there was a significant 44.87% increase in leaf K content recorded over the control under no stress (70 FC). Under drought stress conditions, the control group (0 DAB + 0 CMC) had a leaf K content of 0.31 (%). The addition of 25 C MC treatment resulted in a remarkable 61.29% increase, and when 0.5 DAB treatment was applied during drought stress, there was a 19.35% increase in leaf K content in comparison to the control. Combining 0.5 DAB with 25 CMC led to a substantial 87.10% increase in leaf K content over the control under drought stress. Moreover, 1 DAB treatment showed a 35.48% increase and when treatment 1 DAB was combined with 25 CMC during drought stress, there was a remarkable 112.90% increase in leaf K content contrasting to the control.

### Root potassium, electrolyte leakage, root nitrogen, and root phosphorus

The control group (0 DAB + 0 CMC) had a root K content of 0.57% under 70 FC conditions, while the addition of 25 CMC resulted in a 14.04% increase in root K content (Table 6). The application of 0.5 DAB led to an 8.77% increase and when 0.5 DAB was combined with 25 CMC, there was a significant 26.32% increase in root K content over the control under 70 FC). Similarly, 1DAB treatment showed a 5.26% increase in root K and when 1DAB was combined with 25 CMC, it led to a 21.05% increase in root K content than the control under 70FC conditions. Under drought stress conditions, the control group (0 DAB + 0 CMC) had a root K value of 0.41%. The addition of 25 CMC resulted in a substantial 19.51% increase in root K related to the control under drought stress. The application of 0.5DAB led to a 7.32% increase in root K and when 0.5DAB was combined with 25 CMC, there was a remarkable 26.83% increase in root K related to the control under drought stress. Similarly, 1 DAB treatment showed a 14.63% increase in root K, and when combined with 25 CMC, it led to an impressive 31.71% increase in root K in comparison to the control under drought stress conditions.

**Table 6:** The effect of carboxymethyl cellulose (CMC) and deashed biochar (DAB) on catalase, ascorbate peroxidase (APx), hydrogen peroxide (H_2_O_2_) and malondialdehyde (MDA) of maize cultivated under no drought and drought stress

DAB (%)	Catalase (CAT) (U/mg Protein)		Ascorbate peroxidase (APx) (U/mg Protein)		Hydrogen peroxide (H_2_O_2_) (n mol/g FW)		Malondialdehyde (MDA) (nmol/mg Protein)		Peroxidase (POD) (U/mg Protein)	
	0 CMC	25 CMC	0 CMC	25 CMC	0 CMC	25 CMC	0 CMC	25 CMC	0 CMC	25 CMC
	Field Capacity 70									
0	48.56 ± 1.12c	38.1 ± 1.04e	3.4 ± 0.10b	2.83 ± 0.07d	32.87 ± 0.51c	24.62 ± 1.17e	0.8 ± 0.04b	0.5 ± 0.03e	29.32 ± 0.54c	23.05 ± 1.18e
0.5	41.04 ± 0.88a	27.29 ± 1.38c	2.98 ± 0.04a	2.63 ± 0.05c	27.12 ± 0.53a	13.51 ± 0.81d	0.59 ± 0.02a	0.31 ± 0.04c	26.54 ± 1.27a	16.22 ± 0.62d
1.0	44.44 ± 1.07b	31.3 ± 2.21d	3.06 ± 0.04ab	2.73 ± 0.01c	28.93 ± 0.01b	21.02 ± 1.0d	0.69 ± 0.03a	0.38 ± 0.04d	28.01 ± 0.22b	19.98 ± 1.14de
	Drought Stress									
0	77.34 ± 1.15c	65.66 ± 1.6f	4.94 ± 0.04b	4.04 ± 0.15d	53.3 ± 0.69c	42.26 ± 1.02f	1.44 ± 0.04c	1.11 ± 0.06f	41.85 ± 1.24c	34.73 ± 0.37f
0.5	73.42 ± 1.14b	58.96 ± 2.18e	4.82 ± 0.04a	3.7 ± 0.10d	51.29 ± 0.93b	38.51 ± 1.63e	1.32 ± 0.04b	0.98 ± 0.06e	39.42 ± 1.11b	32.88 ± 0.55e
1.0	70.11 ± 1.39a	52.88 ± 2.21d	4.53 ± 0.16a	3.58 ± 0.03c	47.96 ± 1.71a	35.95 ± 0.84d	1.24 ± 0.03a	0.88 ± 0.03d	36.26 ± 0.90a	31.33 ± 0.66d

Values are mean ± standard deviation (*n* = 3)

In 70FC, the control group with 0 DAB + 0 CMC exhibited a mean electrolyte leakage percentage of 40.27%. When 25 CMC was introduced under 70 FC conditions, there was a 17.54% decrease in electrolyte leakage compared to the control (Table 6). Similarly, applying 0.5 DAB resulted in a 13.50% decrease in electrolyte leakage evaluated to the control, and when combined with 25 CMC, there was a substantial 49.70% decrease in electrolyte leakage under 70 FC. The application of 1 DAB under 70 FC conditions led to an 8.69% decrease in electrolyte leakage, and when combined with 25 CMC, there was a significant 31.00% decrease in electrolyte leakage related to the control. Under drought stress conditions, the control group with 0 DAB + 0 CMC had a mean electrolyte leakage of 58.93%. Introducing 25CMC during drought stress resulted in a 16.00% decrease in electrolyte leakage associated with the control. In contrast, applying 0.5 DAB during drought stress showed only a modest 1.99% decrease in electrolyte leakage in comparison to the control. However, when 0.5 DAB was combined with 25 CMC, there was a substantial 33.84% decrease in electrolyte leakage under drought stress from the control. The use of 1 DAB under drought stress conditions led to a 6.26% decrease in electrolyte leakage, and when combined with 25 CMC, there was a remarkable 38.33% decrease in contrast to the control.

The mean value of the control group (0 DAB + 0 CMC) exhibited a root N value of 0.040 (%). Under no drought (70 FC), all treatments, including 25 CMC, 0.5 DAB, and 1 DAB, exhibited no significant percentage change in root N% compared to the control (Table 6). However, when 0.5 DAB was combined with 25 CMC under 70 FC, there was a noTable 25.00% increase in root N% over the control and a 25.00% increase was observed when 1 DAB and 25 CMV treatment was combined. Under drought stress conditions, the control (0 DAB + 0 CMC) exhibited a root N% of 0.030%. Similarly, the addition of 25 CMC or 0.5 DAB alone did not lead to any significant percentage change in root N% over the control under drought stress. However, when 0.5 DAB was combined with 25 CMC under drought stress, a substantial 33.33% increase in root N% was observed in comparison to the control under drought stress. Additionally, the application of 1DAB treatment showed no specific change or in combination with 25 CMC also resulted in a 33.33% increase in root N% under drought stress over the control.

**Fig. 3:**
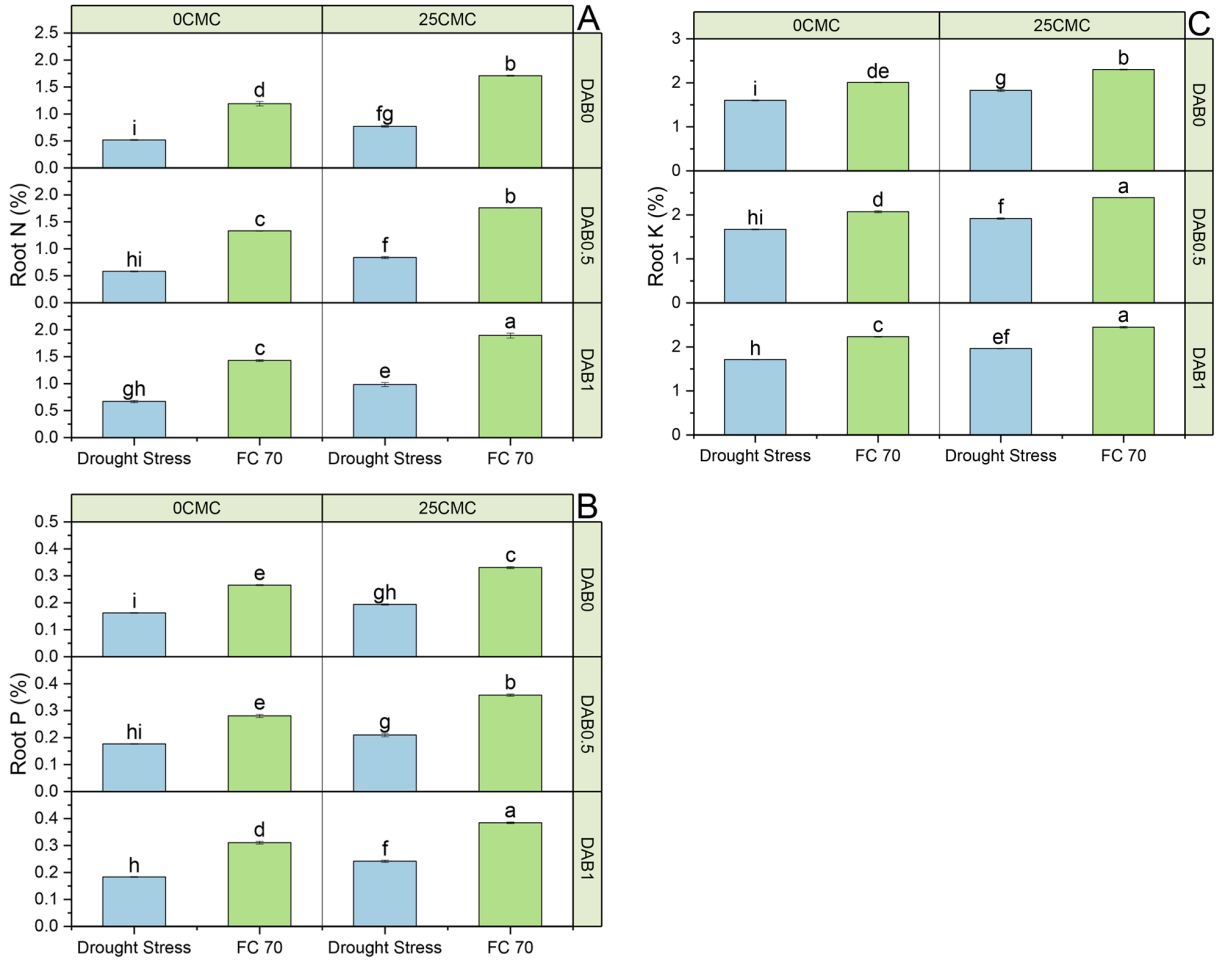
The effect of carboxymethyl cellulose (CMC) and deashed biochar (DAB) roots nitrogen, phosphorus and potassium of maize cultivated under no drought and drought stress. Dots on lines are mean of *n* = 3

Under 70FC conditions with no DAB and CMC (0 DAB + 0 CMC), the mean root P (%) was 0.14%. When 25 CMC was introduced under 70FC conditions, there was a notable increase of 21.43% in root P (%) contrasted to the control (Table 6). Similarly, the application of 0.5 DAB in 70 FC conditions resulted in a 14.29% increase in root P relative to the control, while the combination of 0.5 DAB and 25CMC led to a substantial 42.86% increase in root P assessed to the control. The use of 1 DAB under 70 FC conditions showed a 7.14% increase in root P (%) compared to the control, and when 1 DAB was combined with 25 CMC, there was a significant 28.57% increase in root P. In contrast, under drought stress conditions with no DAB and CMC (0 DAB + 0 CMC), the root P was much lower at 0.04%. However, when 25 CMC was applied under drought stress, there was a remarkable 125.00% increase in root P contrasted to the control. Similarly, the application of 0.5DAB during drought stress resulted in a 25.00% increase in root P, and when combined with 25 CMC, a substantial 175.00% increase in root P was observed over the control. The use of 1 DAB under drought stress conditions showed a 100.00% increase in root P relative to the control, and when 1 DAB was combined with 25 CMC, there was a remarkable 200.00% increase in root P (Fig. 3).

### Peroxidase and superoxidase

Under no stress (70FC), the control group with no DAB or CMC (0 DAB + 0 CMC) exhibited a mean POD activity of 29.32 U/mg protein (Fig. 2). When 25CMC was introduced in the 70FC conditions, there was a decrease of 27.20% in POD activity in comparison to the control. Conversely, the application of 0.5 DAB treatment resulted in a 10.47% decrease in POD activity related to the control under 70 FC. The combination of 0.5 DAB and 25 CMC led to a substantial 80.76% decrease in POD activity under 70FC contrasted to the control. When 1 DAB was used under 70 FC conditions, POD activity decreased by 4.68%, and when 1 DAB was combined with 25CMC, POD activity decreased significantly by 46.75% in comparison to the control. Under drought stress conditions, the control group (0 DAB + 0 CMC) exhibited a higher mean POD activity of 41.85 U/mg protein. The introduction of 25 CMC during drought stress caused a 20.50% decrease in POD activity over the control treatment. Meanwhile, applying 0.5 DAB during drought stress led to a 6.16% decrease in POD activity related to the control. The combination of 0.5 DAB and 25 CMC during drought stress resulted in a 27.28% decrease in POD activity related to the control. Finally, the use of 1 DAB during drought stress conditions showed a 15.42% decrease in POD activity, and when combined with 25 CMC, there was a notable 33.58% decrease in POD activity relative to the control.

In the absence of DAB and CMC (0 DAB + 0 CMC) in a 70 FC, SOD activity was recorded at 19.48 U/mg protein (Fig. 2). The addition of 25 CMC treatment resulted in an 11.76% decrease in SOD activity over the control under 70 FC. When 0.5DAB was introduced, there was a 5.41% decrease in SOD activity evaluated to the control under 70 FC. The combination of 0.5 DAB and 25 CMC exhibited a substantial 34.44% decrease in SOD activity, and the application of 1 DAB led to a significant 1.30% decrease in SOD activity in comparison to the control under 70 FC. However, when 1DAB was combined with 25 CMC as compared to the control in 70 FC, there was a notable 21.30% decrease in SOD activity. Under drought stress conditions, the absence of both DAB and CMC (0 DAB + 0 CMC) led to a SOD activity of 24.80 U/mg protein. The introduction of 25CMC during drought stress resulted in a 12.22% decrease in SOD activity related to the control. When 0.5 DAB was applied, SOD activity decreased slightly by 1.39% over the control under drought stress. Combining 0.5 DAB with 25 CMC exhibited a 15.89% decrease in SOD activity and the application of 1 DAB during drought stress led to a 6.26% decrease in SOD activity in contrast to the control. Additionally, when 1 DAB was combined with 25 CMC during drought stress, there was a significant 19.40% decrease in SOD activity related to the control.

## Catalase activity, ascorbate, hydrogen peroxidase and malondialdehyde

Under non-drought stress conditions (70FC), the control group (0DAB + 0CMC) exhibited a CAT activity of 48.56 U/mg protein. When 25 CMC was introduced, there was a 27.45% decrease in CAT activity from the control under 70FC and the application of 0.5 DAB led to an 18.32% decrease in CAT activity. The most substantial change was observed when both 0.5 DAB and 25 CMC were combined, resulting in a remarkable 77.94% decrease in CAT activity over the control under 70 FC condition. On the other hand, related to the control, when 1DAB was applied under 70FC, there was a 9.27% decrease in CAT activity. When 1DAB was combined with 25CMC, a significant 55.14% decrease in CAT activity was observed compared to the baseline treatment under 70 FC. Under drought stress conditions, the control group (0 DAB + 0 CMC) exhibited a CAT activity of 77.34 U/mg protein. With the addition of 25CMC, there was a 17.79% decrease in CAT activity, and the application of 0.5DAB led to a modest 5.34% decrease in CAT activity in contrast to the control under drought stress. When both 0.5 DAB and 25 CMC were combined, a substantial 31.17% decrease in CAT activity was observed under drought stress over the control. Conversely, when 1 DAB was applied during drought stress, there was a 10.31% increase in CAT activity, and the combination of 1 DAB and 25 CMC showed a notable 46.26% increase in CAT activity under drought stress contrasting to the control.

The addition of 25 CMC treatment led to a 20.14% drop in APx activity under non-drought stress conditions (70 FC), whereas the application of 0.5 DAB treatment showed a 14.09% reduction as compared to the control. When both 0.5 DAB and 25 CMC were combined, there was a substantial 29.28% decrease in APx activity associated with the control in 70FC. However, the use of 1 DAB in the same conditions caused an 11.11% decrease in APx activity, and when combined with 25 CMC, there was a 24.54% decrease related to the control under 70FC. Under drought stress, the control group of APx activity was recorded to be 4.94 U/mg protein. The addition of 25 CMC led to a 22.28% decrease in APx activity, while 0.5 DAB produced only a slight 2.49% decrease in APx activity over the control under drought stress. However, when 0.5 DAB and 25CMC were used together under drought stress, there was a significant 33.51% decrease in APx activity linked to the control. On the other hand, the application of 1 DAB during drought stress resulted in a 9.05% decrease in APx activity, and when combined with 25 CMC, there was a remarkable 37.99% decrease as compared to the control.

Under 70FC, the control group (0 DAB + 0 CMC) had H_2_O_2_ levels at 32.87 nmol/g FW. When 25 CMC was introduced, there was a 33.51% decrease in H_2_O_2_ levels contrasted to the control under 70FC. Similarly, the application of 0.5 DAB resulted in a 21.20% reduction in H_2_O_2_ levels, and when combined with 25 CMC under 70 FC, there was a significant 143.30% decrease in H_2_O_2_ levels associated with the control. In contrast, 1 DAB led to a 13.62% decrease in H_2_O_2_ levels, and when combined with 25 CMC, there was a 56.37% decrease in H_2_O_2_ levels contrasted to the control under 70 FC conditions. Under drought stress conditions, the control group (0DAB + 0CMC) showed H_2_O_2_ levels at 53.30 nmol/g FW. When 25CMC was applied, there was a 26.12% decrease in H_2_O_2_ levels contrasted to the control under drought stress. The use of 0.5 DAB resulted in a 3.92% decrease in H_2_O_2_ levels, while the combination of 0.5 DAB and 25 CMC led to a significant 38.41% decrease in H_2_O_2_ levels over to the control under drought stress. Similarly, 1 DAB resulted in an 11.13% decrease in H_2_O_2_ levels, and when combined with 25 CMC, there was a substantial 48.26% decrease in H_2_O_2_ levels compared to the control under drought stress conditions.

The control group (0 DAB + 0 CMC) had MDA levels of 0.80 nmol/mg protein at 70FC conditions. Under 70 FC conditions, the application of 25 CMC led to a significant 60.00% decrease in MDA levels contrasted to the control. Similarly, when 0.5 DAB was applied under 70 FC conditions, there was a noTable 35.59% reduction in MDA levels over the control under 70FC. The combination of 0.5 DAB and 25 CMC resulted in a 158.06% decrease in MDA levels associated with the control compared to the control under no stress (70 FC). On the other hand, the use of 1 DAB under 70 FC conditions led to a moderate 15.94% decrease in MDA levels, while the combination of 1 DAB and 25 CMC decreased MDA levels by 110.53% relative to the control. Under drought stress conditions, the application of 25 CMC resulted in a 29.73% decrease in MDA levels compared to the control (0 DAB + 0 CMC). When 0.5 DAB was applied during drought stress, there was a slight 9.09% decrease in MDA levels relative to the control. However, when 0.5 DAB was combined with 25 CMC under drought stress, a significant 46.94% decrease in MDA levels was observed over the control. Similarly, the use of 1 DAB under drought stress conditions decreased MDA levels by 16.13%, while the combination of 1 DAB and 25 CMC led to a substantial 63.64% decrease in MDA levels contrasted to the control (Table 6).

### Convex hull, hierarchical cluster analysis

**Fig. 4:**
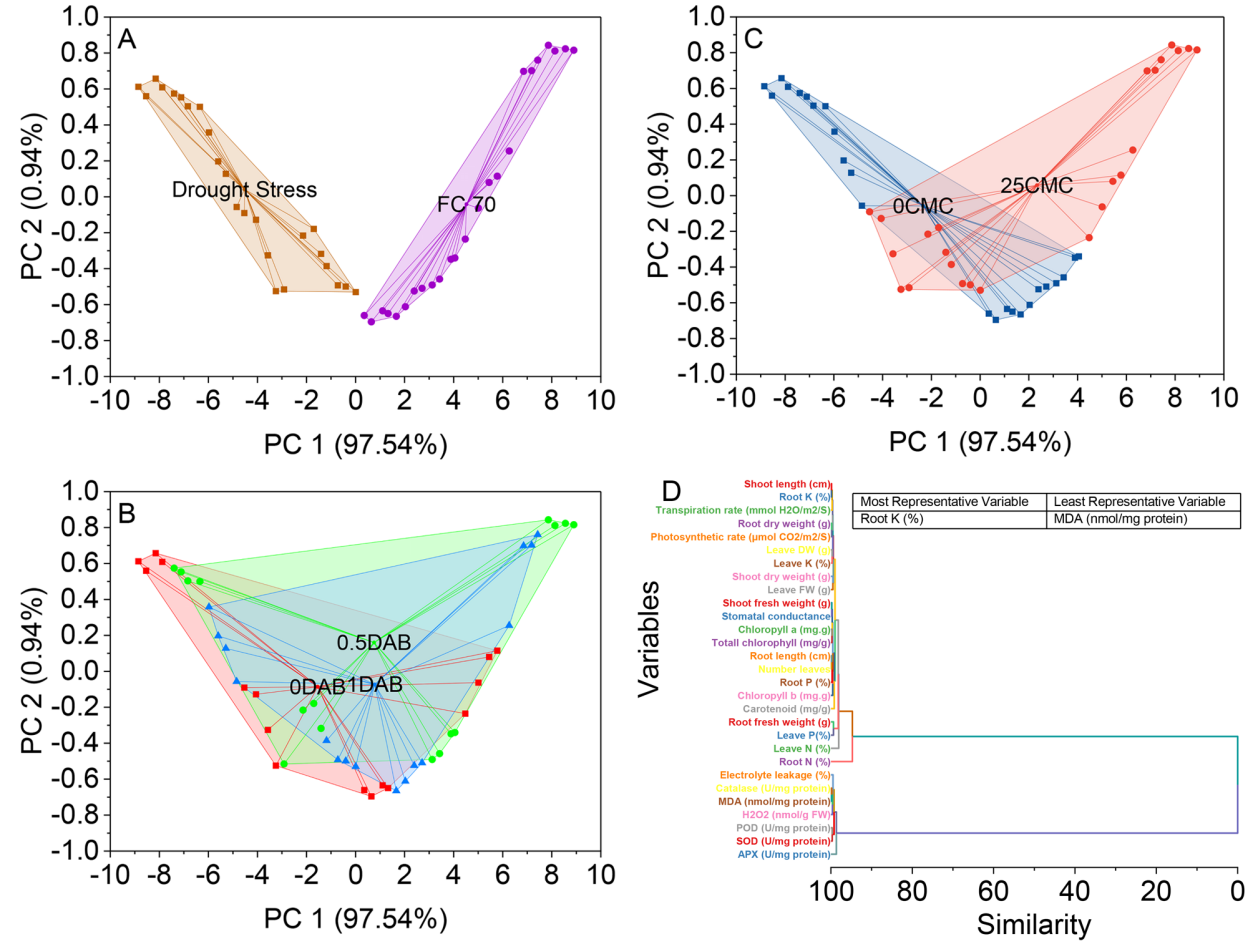
Cluster plot convex hull for stress (70FC) **(A)**, deashed biochar (DAB) **(B)**, carboxymethyl cellulose (CMC) **(C)**, and hierarchical cluster plot **(D)** for studied attributes

The convex hull analysis reveals distinct clusters of data points for the two categories, Drought Stress and 70FC. For Drought Stress, the convex hull spans from a minimum PC 1 value of -8.85218 to a maximum of 0.00823, and from a low PC2 value of -0.53011 to a high of 0.65704. Within this convex hull, 97.54% of the data points fall under the drought stress category. On the other hand, the convex hull for 70FC ranges from a minimum PC1 value of 0.00823 to a maximum of 8.90135, and from a low PC2 value of -0.69592 to a high of 0.82322. Interestingly, all data points associated with 70 FCare enclosed within this convex hull, indicating 100% coverage (Fig. 4A).

The results of the convex hull analysis conducted on the provided dataset are as follows: In the PC1-PC2 space, data points were systematically categorized into distinct Drought Stress levels based on their specific positions within the convex hull. Notably, the analysis found that 97.54% of the data points were situated within the confines of the convex hull associated with the 0 DAB Drought Stress category. Additionally, 0.94% of the data points belonged to this same category, yet they fell outside the convex hull’s boundaries. The assignment of Drought Stress labels to individual data points hinged on their precise coordinates within the convex hull. For example, data points featuring PC1 and PC2 coordinates of -8.85218 and 0.61202, respectively, were categorized as 0 DAB since they were located within the convex hull delineated for this specific Drought Stress level. Likewise, data points exhibiting coordinates such as -5.97378 for PC1 and 0.35776 for PC2 were designated as 1DAB as they were found within the convex hull corresponding to that Drought Stress category (Fig. 4B).

The results of the analysis, which involves convex hull calculations and drought stress scores, are presented in a concise and organized manner. In the dataset, two principal components (PC 1 and PC 2) are associated with drought stress, specifically DAB and CMC. The Drought Stress values are represented as percentages, with PC 1 having a dominance of 97.54% and PC 2 contributing 0.94%. The subsequent section of the results presents scores and associated labels. These scores are numerical values that likely pertain to the analysis. The labels categorize the scores into two main groups, 0 CMC and 25 CMC, which may signify different experimental conditions or states. Within the 0CMC category, scores range from − 8.85218 to 4.05187, with corresponding PC 1 and PC 2 values. It’s evident that these scores represent a specific condition, possibly related to the absence of a certain factor denoted as CMC. On the other hand, the 25 CMC category encompasses scores spanning from − 4.53349 to 8.90135, accompanied by PC 1 and PC 2 values. This suggests an alternate experimental condition or treatment where CMC appears to be present or relevant (Fig. 4C).

Hierarchical cluster analysis was applied to evaluate a dataset featuring two principal components, PC 1 and PC 2, alongside their associated Drought Stress values. The outcome of this analysis unveiled a hierarchical structure of clusters rooted in the similarity between variables. The dataset was segmented into 24 distinctive clusters. It’s worth noting that certain clusters exhibited a consistent level of similarity among their constituent variables, such as Cluster 1 and Cluster 2, boasting relatively low similarity values of 0.15041 and 0.2049, respectively. Conversely, Cluster 23 attracted attention due to its exceptionally high similarity values, indicating a distinctive cluster of variables. Moreover, several clusters, including Cluster 11 and Cluster 14, displayed varying similarity values among their members. Additionally, the analysis identified individual variables that did not form substantial groups with others, such as variable 57, which constituted Cluster 24 independently (Fig. 4D).

### Pearson correlation

**Fig. 5:**
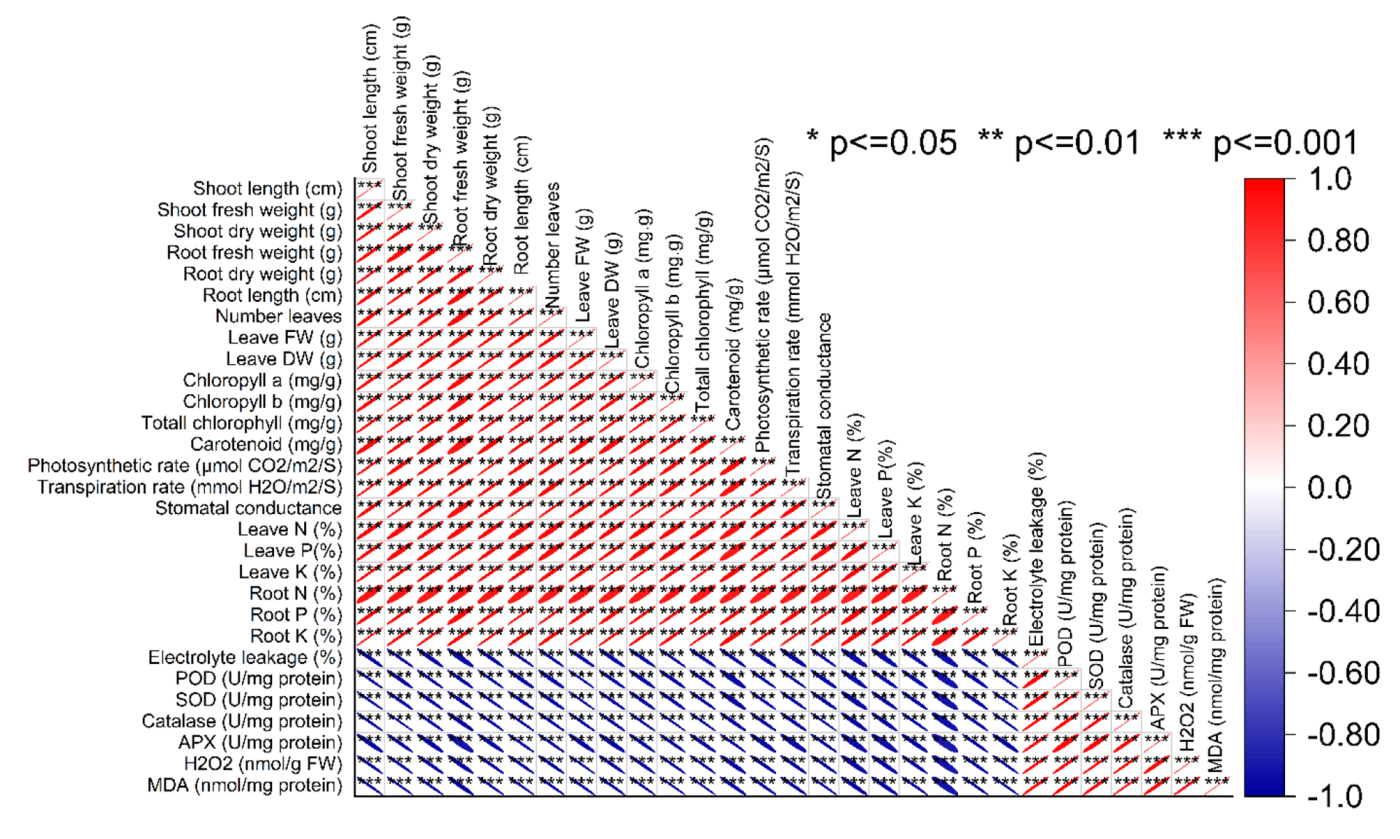
Pearson correlation for studied attributes

The results of the Pearson correlation analysis conducted on various plant growth and physiological parameters reveal significant relationships among these variables (Fig. 4). The correlation matrix provides insights into the strength and direction of these associations. Shoot length (cm) exhibited strong positive correlations with several parameters, including shoot fresh weight (0.98635), shoot dry weight (0.99123), root fresh weight (0.98518), and root dry weight (0.9944), suggesting that longer shoot lengths are associated with higher shoot and root weights. Additionally, shoot length also displayed positive correlations with the number of leaves (0.97879) and various chlorophyll measurements, such as chlorophyll a (0.98738) and chlorophyll b (0.98205), indicating that increased shoot length may coincide with greater chlorophyll content and leaf production. The relationship between shoot fresh weight and shoot dry weight was notably strong (0.98635), illustrating a close correspondence between these two-growth metrics. Furthermore, shoot fresh weight and shoot dry weight exhibited high positive correlations with most other parameters, including root fresh weight, root dry weight, and leaf attributes like leaf fresh weight, leaf dry weight, and total chlorophyll content. Chlorophyll measurements, including chlorophyll a, chlorophyll b, and total chlorophyll, displayed strong positive correlations with one another, with coefficients ranging from 0.98044 to 0.99469, indicating that higher levels of one type of chlorophyll are often associated with increased levels of the others. This suggests a coordinated relationship in chlorophyll production within the plant. Photosynthetic rate (µmol CO2/m2/S) and transpiration rate (mmol H2O/m2/S) exhibited a very strong positive correlation (0.99502), indicating that as photosynthetic rate increases, so does transpiration rate, reflecting the interconnectedness of these processes in plant physiology. In contrast, electrolyte leakage showed negative correlations with most of the parameters, suggesting that higher electrolyte leakage, which can indicate cell damage or stress, tends to coincide with lower values in the measured plant growth and physiological attributes (Fig. 5).

## Discussion

The study delved into various physiological markers, including chlorophyll content, carotenoid presence, photosynthetic activity, transpiration rate, stomatal conductivity, and electrolyte leakage. Both CMC and DAB treatments positively influenced these traits, suggesting heightened photosynthesis efficiency, potentially linked to increased nutrient uptake and improved soil water availability facilitated by these amendments.

The decrease in stomatal conductance and transpiration rate indicated a potential reduction in water loss, particularly crucial under drought-stress conditions (Table 5) [35]. This adaptive response enables the plant to conserve water, sustain turgor pressure, and allocate more resources to growth and stress tolerance mechanisms, particularly in drought-stressed scenarios [36].

The research also provided valuable insights into the biochemical and physiological responses of maize plants to field capacity and drought stress [37]. Under optimal field conditions, enzymatic activities and oxidative stress parameters remained balanced (Table 6), reflecting a well-regulated antioxidant defense mechanism [38]. However, during drought stress, significant changes in enzymatic activity and oxidative stress indicators were observed, illustrating the plant’s adaptive responses to water scarcity.

The increased activity of antioxidant enzymes like peroxidase (POD) [39], superoxide dismutase (SOD), catalase, and ascorbate peroxidase (APX) indicated the plant’s efforts to mitigate oxidative stress caused by elevated reactive oxygen species (ROS) levels during drought stress (Table 6). The study highlighted the importance of these mechanisms in protecting cellular components from damage [40].

Furthermore, the research emphasized the need to comprehend these physiological responses when developing strategies to enhance plant resilience in drought-prone environments. The findings suggested that the use of CMC and DAB could significantly improve maize development and physiological responses, particularly in drought-stressed conditions, by enhancing soil water retention, improving soil structure, increasing nutrient availability, and reducing water loss [41].

The practical implications of these findings include the potential to address challenges posed by water constraints and changing climatic conditions in maize cultivation, thereby contributing to sustainable agriculture [42, 43]. However, the study also underscored the importance of further research to explore the long-term consequences and environmental implications of these agricultural changes, with a call for additional investigation into specific genes and processes involved in these actions [42, 43].

## Conclusion

In conclusion, the use of 1% DAB + 25mM CMC exhibits the capacity to enhance the growth attributes of maize when exposed to drought conditions. The incorporation of 1% DAB + 25mM CMC has proved its capacity to enhance the uptake of essential nutrients such as nitrogen (N), phosphorus (P), and potassium (K) in both the root and shoot. This nutrient enhancement significantly contributes to the overall improvement of maize growth during drought-stress conditions. Furthermore, the 1% DAB + 25 mM CMC treatment exhibits the potential to regulate antioxidants under drought stress, thereby potentially mitigating the adverse effects of drought on maize. Further research at the field level is recommended to validate 1% DAB + 25mM CMC as the optimal treatment for alleviating drought stress in maize.

## Data Availability

All data generated or analysed during this study are included in this published article.
